# Metal-halide perovskites for high-efficiency radiation shielding applications

**DOI:** 10.1038/s41377-022-01060-8

**Published:** 2023-01-02

**Authors:** Qingfeng Dong, Yanjun Fang

**Affiliations:** 1 State Key Laboratory of Supramolecular Structure and Materials, College of Chemistry, Jilin University, Changchun, 130012 China; 2 State Key Laboratory of Silicon Materials and School of Materials Science and Engineering, Zhejiang University, Hangzhou, 310027 China

**Keywords:** Optoelectronic devices and components, Photonic devices

## Abstract

The ionizing radiation possesses extremely strong penetration capability, which poses serious risk on the health of the human body and jeopardize electronics. Here the authors demonstrate that MAPbI_3_/epoxy composites prepared by a simple method show high radiation shielding performance.

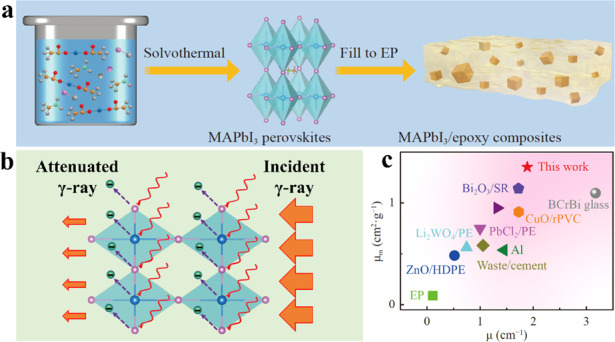

## Abstract (web summary)

The MAPbI_3_/epoxy composite was prepared by a simple solvothermal method and show high radiation shielding performance.

The ionizing radiation, including X-ray and gamma-ray, possesses extremely strong penetration capability, which poses serious risk on the health of the human body due to its damage to deoxyribonucleic acid that would profoundly increase the probability of developing cancer diseases^1,2^. Meanwhile, the ionizing radiation could also jeopardize the efficacy and stability of electronic devices when operated in the environment with high-dose radiation exposure, like in the space or nuclear plants^3–5^. Therefore, the high-performance radiation shielding materials are urgently required to alleviate the radiation risks.

The key parameter that determines the attenuation power of radiation shielding materials is their linear attenuation coefficients, which is correlated with the atomic number of the active materials^6,7^. Consequently, materials with large atomic number, such as lead, concretes, tungsten, etc., have been widely adopted for the shielding of ionizing radiation^8,9^. Nevertheless, in some application scenarios like in space environment or medical diagnosis, the radiation shielding materials with both large linear attenuation coefficient and small density are more favorable since the portability and flexibility of the radiation protection can be greatly enhanced^10^. Recently, metal halide perovskites (MHPs) have gained increasing research attention as the next-generation radiation detection materials due to their low-cost solution processibility, excellent charge transport properties, as well as strong radiation-stopping power^11–14^. In view of the large atomic number and the resultant high mass attenuation coefficient, the MHPs can potentially also function as the radiation shielding materials, which, however, has seldom been explored.

A recent publication in *Light: Advanced Manufacturing* by Cui et al. provide a new insight into the engineering application of classic MHPs as high-performance radiation shielding materials^15^. They prepared MAPbI_3_/epoxy composites by a simple method with high radiation shielding performance against 59.5 keV gamma ray (Fig. 1a, b). High linear attenuation coefficient (1.887 cm^−1^) and mass attenuation coefficient (1.352 cm^2^/g) achieved in prepared MAPbI_3_/epoxy composites, which show better gamma ray (59.5 keV) shielding ability in terms of the larger μ and μ_m_ than the most commonly used shielding materials (Fig. 1c).

**Fig. 1:**
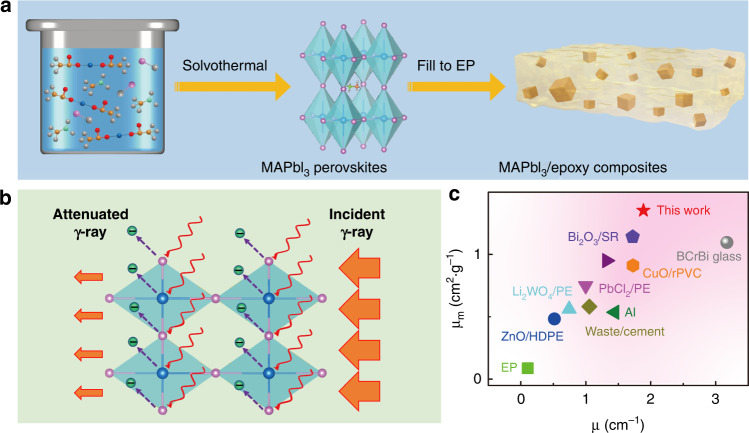
MAPbI_3_/epoxy based radiation shielding materials. **a** Schematic illustration of the MAPbI_3_ microcubic crystals and the MAPbI_3_/epoxy composites; **b** Schematic illustration of gamma ray shielding of the MAPbI_3_/epoxy composites; **c** μ and μ_m_ of some commonly used materials reported in recent years.

Despite the high linear attenuation coefficient and mass attenuation coefficient achieved in the MAPbI_3_/epoxy composite, more works remains to be done in the future to promote its practical application as the radiation shielding materials. For instance, the long-term stability of the MAPbI_3_/epoxy composite, including the moisture, irradiation, and temperature stability, needs to be evaluated to accommodate the harsh environments. Besides, future attempts can be done to replace MAPbI_3_ with lead-free MHPs for reduced environmental toxicity. Nevertheless, the presented work demonstrated the bright future of MHPs/epoxy composite as the next-generation high-performance yet low-cost radiation shielding materials.

## References

[1] Brenner DJ (2001). Estimated risks of radiation-induced fatal cancer from pediatric CT. Am. J. Roentgenol..

[2] Lin EC (2010). Radiation risk from medical imaging. Mayo Clin. Proc..

[3] Benton ER, Benton EV (2001). Space radiation dosimetry in low-Earth orbit and beyond. Nucl. Instrum. Methods Phys. Res. Sect. B: Beam Interact. Mater. At..

[4] Mukherjee B (2016). Near space radiation dosimetry in Australian outback using a balloon borne energy compensated PIN diode detector. Radiat. Meas..

[5] Tu YG (2021). Perovskite Solar Cells for Space Applications: Progress and Challenges. Adv. Mater..

[6] Wu HD (2021). Metal Halide Perovskites for X-Ray Detection and Imaging. Matter.

[7] Wei HT, Huang JS (2019). Halide lead perovskites for ionizing radiation detection. Nat. Commun..

[8] Mahmoud ME (2021). Ceramic tiles doped with lead oxide nanoparticles: Their fabrication, physical, mechanical characteristics and γ-ray shielding performance. Radiat. Phys. Chem..

[9] Mahmoud ME (2020). Investigation of physical, mechanical and gamma-ray shielding properties using ceramic tiles incorporated with powdered lead oxide. Ceram. Int..

[10] More CV (2021). Polymeric composite materials for radiation shielding: a review. Environ. Chem. Lett..

[11] Dong QF (2015). Electron-hole diffusion lengths > 175 μm in solution-grown CH3NH3PbI3 single crystals. Science.

[12] Liu FZ (2022). Recent Progress in Halide Perovskite Radiation Detectors for Gamma-Ray Spectroscopy. ACS Energy Lett..

[13] He YH (2021). CsPbBr_3_ perovskite detectors with 1.4% energy resolution for high-energy γ-rays. Nat. Photonics.

[14] Song YL (2021). Elimination of Interfacial-Electrochemical-Reaction-Induced Polarization in Perovskite Single Crystals for Ultrasensitive and Stable X-Ray Detector Arrays. Adv. Mater..

[15] Cui, K. et al. Crystal plane engineering of MAPbI_3_ in epoxy-based materials for superior gamma-ray shielding performance. *Light Adv. Manuf.* **3**, 51 (2022).

